# Bone morphogenetic protein 2 inhibits growth differentiation factor 8-induced cell signaling via upregulation of gremlin2 expression in human granulosa-lutein cells

**DOI:** 10.1186/s12958-021-00854-6

**Published:** 2021-11-27

**Authors:** Xiaoyan Luo, Hsun-Ming Chang, Yuyin Yi, Yingpu Sun, Peter C. K. Leung

**Affiliations:** 1 Center for Reproductive Medicine, The First Affiliated Hospital of Zhengzhou University, 40, Daxue Road, Zhengzhou, 450052 Henan China; 2 Department of Obstetrics and Gynaecology, BC Children’s Hospital Research Institute, University of British Columbia, Room 317, 950 West 28th Avenue, Vancouver, BC V5Z 4H4 Canada; 3 Henan Key Laboratory of Reproduction and Genetics, The First Affiliated Hospital of Zhengzhou University, Zhengzhou, China; 4 Henan Provincial Obstetrical and Gynecological Diseases (Reproductive Medicine) Clinical Research Center, Zhengzhou, China

**Keywords:** BMP2, GREM1, GREM2, GDF8, Human granulosa cells

## Abstract

**Background:**

Bone morphogenetic protein 2 (BMP2), growth differentiation factor 8 (GDF8) and their functional receptors are expressed in human ovarian follicles, and these two intrafollicular factors play essential roles in regulating follicle development and luteal function. As BMP antagonists, gremlin1 (GREM1) and gremlin2 (GREM2) suppress BMP signaling through blockage of ligand-receptor binding. However, whether BMP2 regulates the expression of GREM1 and GREM2 in follicular development remains to be determined.

**Methods:**

In the present study, we investigated the effect of BMP2 on the expression of GREM1 and GREM2 and the underlying mechanisms in human granulosa-lutein (hGL) cells. An established immortalized human granulosa cell line (SVOG) and primary hGL cells were used as study models. The expression of GREM1 and GREM2 were examined following cell incubation with BMP2 at different concentrations and time courses. The TGF-β type I inhibitors (dorsomorphin, DMH-1 and SB431542) and small interfering RNAs targeting ALK2, ALK3, SMAD2/3, SMAD1/5/8 and SMAD4 were used to investigate the involvement of the SMAD-dependent pathway.

**Results:**

Our results showed that BMP2 significantly increased the expression of GREM2 (but not GREM1) in a dose- and time-dependent manner. Using a dual inhibition approach combining kinase inhibitors and siRNA-mediated knockdown, we found that the BMP2-induced upregulation of GREM2 expression was mediated by the ALK2/3-SMAD1/5-SMAD4 signaling pathway. Moreover, we demonstrated that BMP2 pretreatment significantly attenuated the GDF8-induced phosphorylation of SMAD2 and SMAD3, and this suppressive effect was reversed by knocking down GREM2 expression.

**Conclusions:**

Our findings provide new insight into the molecular mechanisms by which BMP2 modulates the cellular activity induced by GDF8 through the upregulated expression of their antagonist (GREM2).

## Introduction

Originally identified due to its pivotal role in bone formation and development, bone morphogenetic protein 2 (BMP2) was the first member of the BMP subfamily to be discovered [1]. In addition to the skeletal system, recent studies have demonstrated the crucial roles of BMP2 in regulating multiple physiological functions, including female reproduction. BMP2 and its functional receptors are expressed in human ovarian follicles and the corpus luteum, and this intrafollicular factor plays an essential role in regulating follicle development and luteal function [2]. Specifically, BMP2 stimulates primordial follicle formation by promoting the differentiation of germ cells to oocytes and somatic cells to pregranulosa cells in the hamster ovary [3]. Additionally, BMP2 promotes follicular growth by upregulating the expression of follicle-stimulating hormone (FSH) receptor and estradiol production in human granulosa cells [4, 5]. Moreover, BMP2 is an anti-luteinization factor because of its ability to suppress the expression of luteinizing hormone (LH) receptor and progesterone production [4, 5]. Our previous studies also demonstrated that BMP2 interacts with other intrafollicular factors and modulates cell–cell communication and cumulus expansion in human granulosa-lutein (hGL) cells [5–9]. Evidence obtained from clinical studies indicated that BMP2 is also a potential indicator of oocyte maturation and fertilization [10]. In modern nomenclature, growth and differentiation factor 8 (GDF8, also known as myostatin) is also a member of the BMP subfamily [2]. GDF8 is a potent negative regulator of skeletal muscle growth and development, as the targeted depletion of *Gdf8* in mice results in muscle fiber hypertrophy and hyperplasia [11, 12]. In addition to its crucial role in the musculoskeletal system, GDF8 is expressed in the ovaries of chicken [13], cattle [14], and humans [2]. Our previous studies have shown a novel role for GDF8 in upregulating various follicular functions during folliculogenesis [15–20]. Similar to other members of the transforming growth factor β (TGF-β superfamily), BMPs/GDFs initiate their cellular activity by binding to type I and type II serine/threonine kinase receptors. Upon ligand-receptor binding, the type I receptors are phosphorylated in the highly conserved juxta-membrane region known as the Gly-Ser (GS) domain, which then triggers downstream signaling pathways and regulate target gene transcription in the cell nucleus [10]. BMP signaling activity is extensively regulated at multiple levels, including extracellular modulation by antagonists through direct binding, intracellular modulation by blocking R-SMAD phosphorylation via inhibitory SMADs (SMAD6 and SMAD7), and membrane modulation via pseudoreceptors such as BAMBI [21]. Extracellular BMP antagonists contain cystine knots, by which these antagonists compete with BMP receptors and directly bind to BMP ligands. As members of the differential screening-selected gene in neuroblastoma (DAN) family, gremlin1 (GREM1) and gremlin2 (GREM2) are well-known extracellular BMP antagonists that form highly stable noncovalent dimers [22]. In cultured mouse granulosa cells, the addition of either GREM1 or GREM2 significantly antagonizes the BMP4-induced suppression of progesterone production [23]. The antagonistic effect of these two antagonists varies in different cells. Compared with GREM1, GREM2 has a broad and strong inhibitory effect on SMAD signaling induced by BMP2 and BMP4, whereas both GREM1 and GREM2 have no antagonistic effect on the reporter activity induced by activin, TGF-β, GDF5 and GDF9 in 293 T cells [23]. To date, whether GREM1 or GREM2 has an antagonistic effect on other members of the TGF-β superfamily, such as GDF8, in human granulosa cells has never been reported. GREM1 and GREM2 are expressed in the ovaries of mice [11] and humans [24], and these two factors play functional roles in regulating embryological ovarian and follicular development, and several reproductive disorders, such as polycystic ovary syndrome [25], diminished ovarian reserve [26], endometriosis [27], and endometrial cancer [28]. Global GREM1 knockout mice die within 48 h after birth with an abnormal ovarian phenotype: a decreased number of oocytes and delayed primordial follicular development [29]. Elevated expression of GREM1 in cumulus cells is associated with oocyte maturity, embryonic development, and pregnancy outcome in women undergoing in vitro fertilization (IVF) [30, 31]. GREM2 (but not GREM1) is expressed in primordial and early-stage developing follicles, and GREM2 participates in regulating the primordial to primary follicle transition [24, 32].

In the ovary, the expression of GREM1 and GREM2 is regulated by gonadotropins. In mouse ovaries, GERM2 is upregulated by PMSG and downregulated by HCG [23]. In addition to gonadotropins, a variety of locally produced growth factors, such as TGF-β1 and BMP7, have been shown to promote the expression of GREM1 in humans and mice [33–35]. To date, whether the expression of GREM1 and GREM2 can be regulated by BMP2 in human granulosa cells remains to be elucidated. In the present study, we sought to investigate the effect of BMP2 on the regulation of its antagonists (GREM1 and GREM2) and the underlying molecular mechanisms. Furthermore, we also examined the antagonistic effect of GREM2 on BMP2 and GDF8 in hGL cells.

## Materials and methods

### Cell culture

All the experiments were carried out after approval by the University of British Columbia Research Ethics Board. In the present study, we used a non-tumorigenic immortalized human granulosa cell line, SVOG cells, which was previously produced by transfecting primary hGL cells with the SV40 large T antigen [36] as the cell model. SVOG cells have been used to study the biological functions and molecular mechanisms of human granulosa cells [37–39]. Primary hGL cells were isolated from the follicle fluid of the patients undergoing IVF treatment with informed consent as previously described [16, 40]. Briefly, SVOG cells and primary hGL cells were seeded at a density of 5 × 10^5^ cells per well in 6-well plates and 2 × 10^5^ cells per well in 12-well plates respectively. All the cells were cultured in Dulbecco’s modified Eagle’s medium/nutrient mixture F-12 (DMEM/F-12) medium (Sigma–Aldrich Corp., Oakville, ON) supplemented with 10% charcoal/dextran-treated fetal bovine serum (HyClone, Logan, UT), 100 U/mL penicillin (Invitrogen, Life Technologies, Carlsbad, CA), 100 μg/mL streptomycin sulfate (Invitrogen) and 1 × GlutaMAX (Invitrogen) in a humidified atmosphere containing 5% CO_2_ at 37 °C. The culture medium was changed every 48 h. Serum starvation was induced by culturing cells in serum-free medium for 18–24 h before specific treatment in all experiments.

### Reagents and antibodies

Recombinant human BMP2 Protein, Recombinant human GDF8 Protein, Recombinant human GREM2 Protein, dorsomorphin dihydrochloride (DM) and dorsomorphin homolog 1 (DMH-1; 4-[6-[4-(1-methylethoxy) phenyl] pyrazole [1, 5-a] pyrimidin-3-yl]-quinoline) were obtained from R&D Systems (Minneapolis, MN). SB431542 was purchased from Sigma–Aldrich. Rabbit polyclonal anti-phospho-SMAD1 (Ser463/465)/SMAD5 (Ser463/465)/SMAD8 (Ser426/428) (#13820), rabbit monoclonal anti-phospho-SMAD2 (Ser465/467; 138D4, diluted at 1:1000), mouse monoclonal anti-SMAD2 (L16D3, diluted at 1:1000), rabbit monoclonal anti-phospho-SMAD3 (Ser423/425; C25A9, diluted at 1:1000), rabbit monoclonal SMAD3 (C67H9, diluted at 1:1000), and rabbit polyclonal anti-SMAD4 (catalog no. 9515, diluted at 1:1000) antibodies were obtained from Cell Signaling Technology (Danvers, MA). Rabbit polyclonal anti-SMAD1/5/8 (N-18; sc-6031-R) and mouse monoclonal anti-GAPDH antibodies (sc-47,724) were obtained from Santa Cruz Biotechnology (Santa Cruz, CA). Horseradish peroxidase-conjugated goat anti-rabbit IgG and goat anti-mouse IgG were purchased from Bio–Rad Laboratories (Hercules, CA).

### Real-time RT-qPCR

After treatment, the cells were washed with cold phosphate-buffered saline (PBS), and total RNA was extracted with TRIzol reagent (Invitrogen, Life Technologies) according to manufacturer’s instructions. A total of 2 μg RNA was reverse transcribed into first-strand cDNA with random primers and Moloney murine leukemia virus (MMLV) reverse transcriptase (Promega, Madison, WI) in a final volume of 20 μL. SYBR Green or TaqMan RT-qPCR was performed using an Applied Biosystems 7300 Real-Time PCR System. Each 20 μL SYBR Green qPCR reaction contained 10 μL of 2 × SYBR Green PCR Master Mix (Applied Biosystems, Foster City, California), 20 ng cDNA, and 250 nM of each specific primer. The primer sequences used in this study were as follows: *GREM1*: 5′-TCATCAACCGCTTCTGTTACG-3′ (sense) and 5′-GGCTGTAGTTCAGGGCAGTT-3′ (antisense); *GREM2*: 5′-ATCCCCTCGCCTTACAAGGA-3′ (sense) and 5′-TCTTGCACCAGTCACTCTTGA-3′ (antisense); *GAPDH*: 5′-GAGTCAACGGATTTGGTCGT-3′ (sense) and 5′-GACAAGCTTCCCGTTCTCAG-3′(antisense); *SMAD2*: 5′-GCCTTTACAGCTTCTCTGAACAA-3′ (sense) and 5′-ATGTGGCAATCCTTTTCGAT-3′ (antisense); *SMAD3*: 5′-CCCCAGCACATAATAACTTGG-3′ (sense) and 5′-AGGAGATGG AGCACCAGAAG-3′ (antisense); and *SMAD4*: 5′-TGGCCCAGGATCAGTAGGT-3′ (sense) and 5′-CATCAACACCAATTCCAGCA-3′ (antisense); TaqMan gene expression assays for *ALK2* (Hs00153836_m1), *ALK3* (Hs01034913_g1), *SMAD1* (Hs01077084_m1), *SMAD5* (Hs00195437_m1), SMAD8 (Hs00195441_m1), and *GAPDH* (catalog no. Hs02758991_g1) were purchased from Applied Biosystems. Each 20 μL TaqMan qPCR reaction contained 10 μL 2 × TaqMan Gene Expression Master Mix (Applied Biosystems), 20 ng cDNA, and 1 × specific TaqMan assay containing primers and probe. The specificity of each assay was validated by dissociation curve analysis and agarose gel electrophoresis of PCR products. All experiments were performed for at least three times, and each sample was loaded in triplicate. *GAPDH* was used as the reference gene, and the relative mRNA levels of target genes were quantified using the comparative Ct method (2^–∆∆Ct^).

### Small interfering RNA transfection

When cells were 50–60% confluent, we transfected them for 48 h with 25 nM ON-TARGETplus Nontargeting Control Pool siRNA or ON-TARGETplus SMART Pool siRNA (Dharmacon, GE Healthcare Life Sciences) targeting ALK2, ALK3, SMAD2, SMAD3, SMAD1, SMAD5, SMAD8, SMAD4, or GREM2 using Lipofectamine RNAiMAX (Life Technologies) according to the manufacturer’s instructions as previously described [41]. RT-qPCR or western blot analysis was carried out to confirm the knockdown efficiency of each siRNA.

### Western blot analysis

The protocol of the western blot analysis was based on our previous study [42]. Briefly, after specific treatment, cells were washed 3 times with cold PBS and lysed in ice-cold lysis buffer supplemented with a protease inhibitor cocktail (Sigma–Aldrich) for 30 min on ice. The extracts were centrifuged at 12,000×g for 15 min at 4 °C to remove cellular debris. Protein concentrations were measured using the DC Protein Assay (Bio–Rad Laboratories) with bovine serum albumin (BSA) as the standard. A total of 30 μg protein from each sample was loaded and separated by 10% sodium dodecyl sulfate polyacrylamide gel electrophoresis and transferred onto the polyvinylidene difluoride (PVDF) membranes for 90 min. The membranes were blocked with 5% nonfat dry milk for 1 h at room temperature in tris-buffered saline containing 0.1% Tween 20 (TBST) and incubated with specific primary antibodies at 4 °C overnight. The next day, membranes were washed 3 times with TBST and incubated with appropriate secondary antibodies (1:5000 in TBST) for 1 h at room temperature. The immunoreactive bands were detected using an enhanced chemiluminescent substrate or a SuperSignal West Femto Chemiluminescence Substrate (Pierce, Rockford, IL, USA) followed by exposure to CL-XPosure film (Thermo Fisher, Invitrogen). When required, membranes were stripped with stripping buffer (62.5 mM Tris-HCl [pH 6.8], 100 mM β-mercaptoethanol, and 2% (wt/vol) SDS) at 50 °C for 30 min and reprobed with antibodies against GAPDH, SMAD 1/5/8, SMAD2, or SMAD3. The films were scanned and the protein expression levels were quantified with ImageJ software.

### Measurement of the protein levels of GREM2

After specific treatments, cell proteins were harvested using the same approach used for western blot analysis. A human GREM2 ELISA kit (MBS8248357, Mybiosource, San Diego, CA) was used to detect the protein levels of GREM2 according to the manufacturer’s instructions. The inter- and intra-assay coefficients of variation for the assays were < 7%. Each sample was measured in triplicate, and the results are presented as the fold change relative to the control.

### Statistical analysis

Three independent replicated experiments were performed with at least three culture plates per experiment. The results were analyzed by one-way ANOVA followed by Tukey’s multiple-comparison tests using PRISM software (GraphPad Software, San Diego, CA). All the data are presented as the mean ± SEM. A *P* < 0.05 was considered significantly different.

## Results

### BMP2 upregulates the expression of GREM2 in SVOG cells

**Fig. 1:**
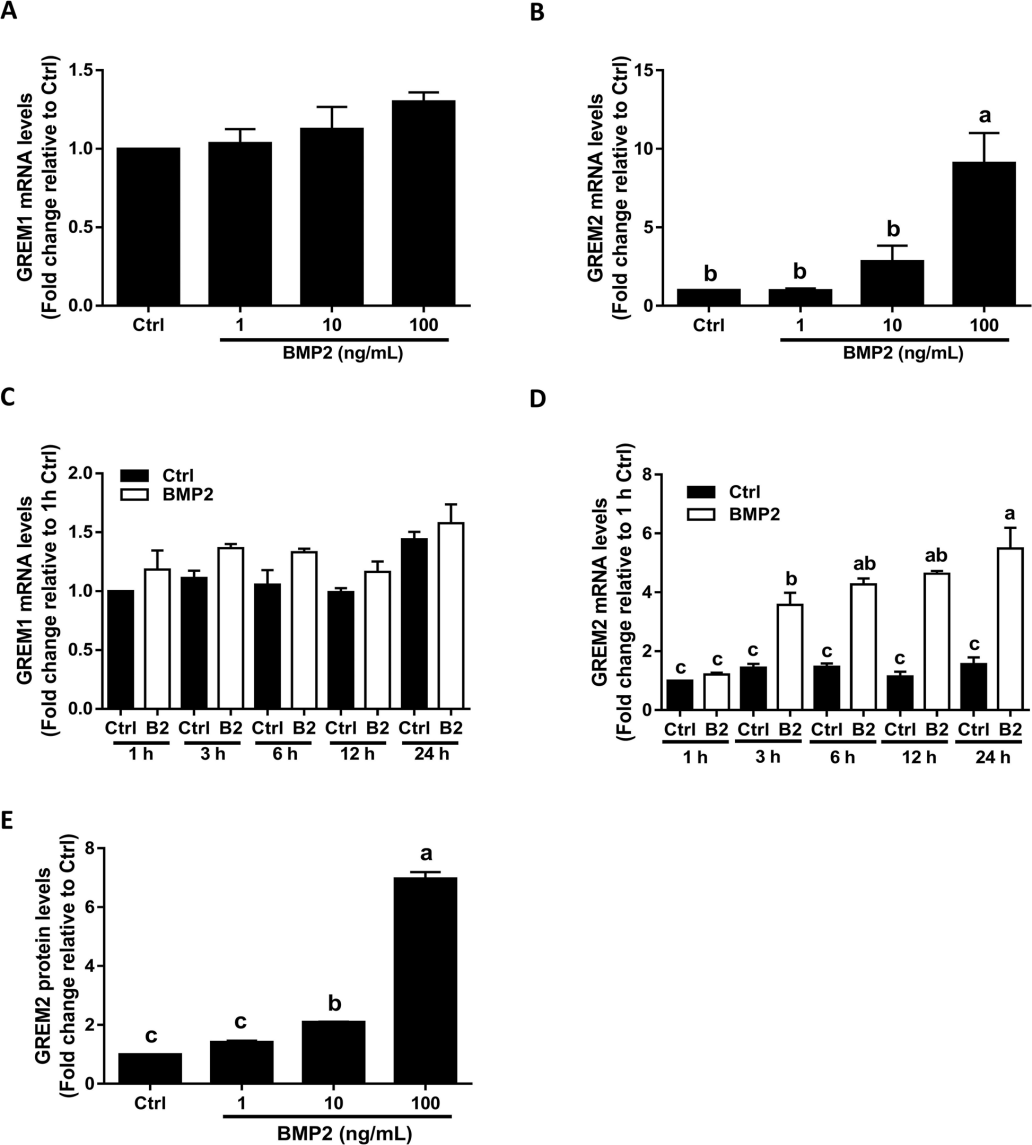
Effects of BMP2 on the expression of GREM1 and GREM2 in immortalized human granulosa-lutein (SVOG) cells. **A** and **B**, the concentration-dependent effects of BMP2 on the mRNA levels of GREM1 (**A**) and GREM2 (**B**). **C** and **D**, and the tome-course seffects of BMP2 on the mRNA levels of GREM1 (**C**) and GREM2 (**D**). **E**, the concentration-dependent effects of BMP2 on the protein levels of GREM2. **F**, Immunostaining showing the effect of BMP2 on the localization of GREM2. The results are expressed as the mean ± SEM of at least three independent experiments, and values without common letters are considered significantly different (*P* < 0.05). Letters are used to denote the statistically significant differences between variables. If the letters on the column bar of two groups are the same (E.g. “a” vs. “a” or “b” vs. “b”), it means that there is no statistical difference between two groups. On the other hand, if the letters on the column bar of two groups are different (E.g. “a” vs. “b” or “b” vs. “c”), it means that there is significant difference between two groups*.* BMP2, bone morphogenetic protein 2; Ctrl, control; GREM1, gremlin1; GREM2, gremlin2

To investigate the effects of BMP2 on the expression of GREM1 and GREM2, we first treated SVOG cells with a vehicle control (Ctrl) or different concentrations (1, 10, or 100 ng/ml) of BMP2 for 12 h. The doses of BMP2 that were used in this study were based on previous clinical studies showing that the concentrations of BMP2 in human follicular fluid range from 1 to 115 ng/ml (with an average of 45–55 ng/ml) [10]. The results showed that BMP2 treatment did not affect the mRNA levels of GREM1 (Fig. 1A). However, treatment with BMP2 significantly increased the mRNA level of GREM2 in a concentration-dependent manner (Fig. 1B). Similarly, the time-course study showed that BMP2 treatment (50 ng/ml) did not affect the mRNA levels of GREM1 at any of the time points examined (1, 3, 6, 12, and 24 h) (Fig. 1C). However, BMP2 significantly increased the mRNA levels of GREM2 starting at 3 h, and the effect persisted until 24 h after treatment (Fig. 1D). Additionally, the protein levels of GREM2 were measured using ELISA, and the results showed that BMP2 treatment increased the protein levels of GREM2 in a concentration-dependent manner (Fig. 1E).

### ALK2 and ALK3 mediate the BMP2-induced upregulation of GREM2 in SVOG cells

**Fig. 2:**
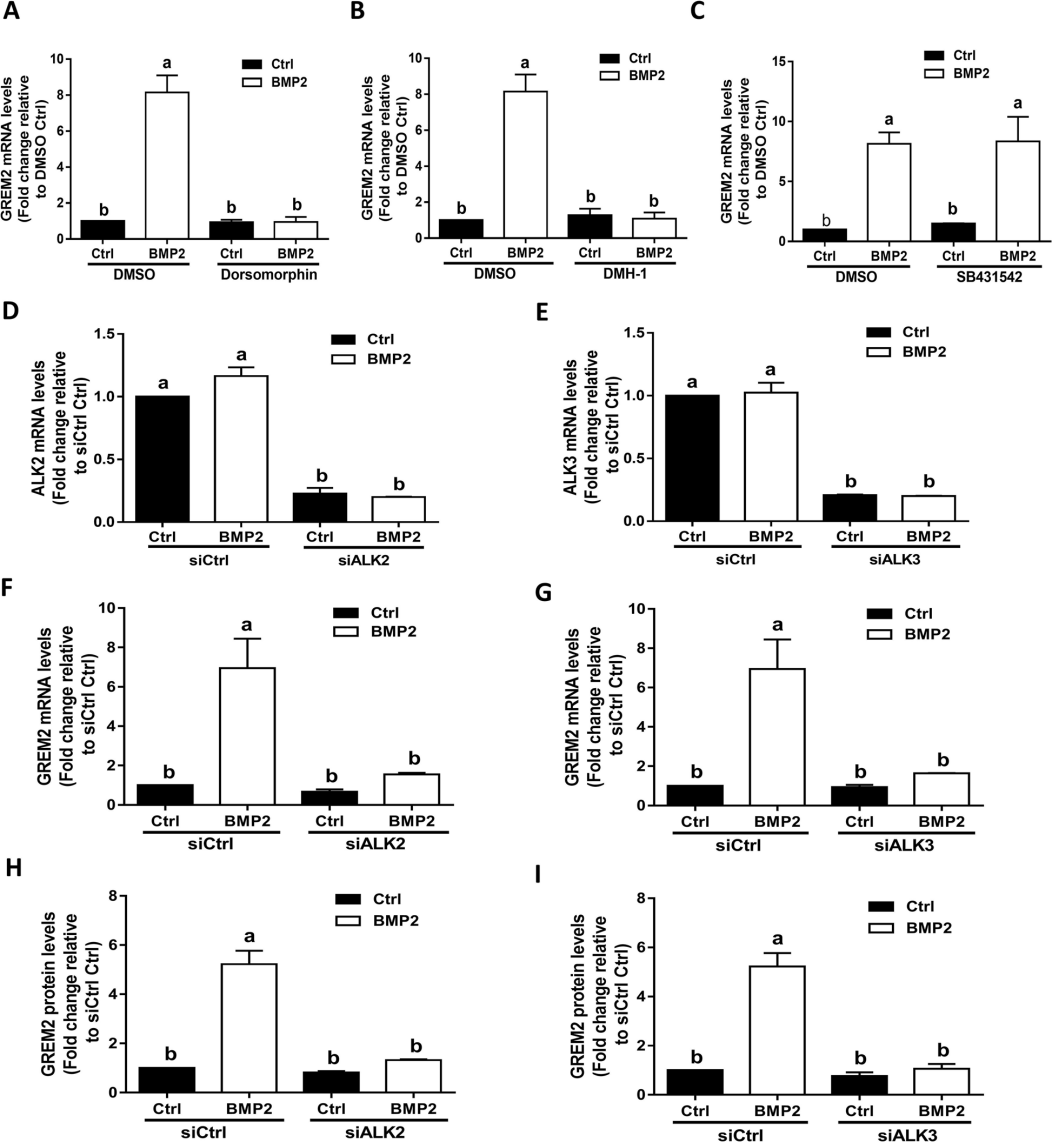
ALK2 and ALK3 mediate the BMP2-induced upregulation of GREM2 in SVOG cells. **A**, **B**, and **C**, the effects of the TGF-β type I receptor inhibitors 10 μM dorsomorphin (**A**), 10 μM DMH-1 (**B**), or 10 μM SB431542 (**C**) on the BMP2-induced increase in the mRNA level of GREM2. **D**, **F**, and **H**, the effects of knockdown of ALK2 on the BMP2-induced increases in the expression of GREM2. E, G, and I, the effects of knockdown of ALK3 on the BMP2-induced increases in the expression of GREM2. The results are expressed as the mean ± SEM of at least three independent experiments, and values without common letters are considered significantly different (*P* < 0.05)

Similar to other TGF-β superfamily members, BMPs exert their biological functions by binding to type I and type II receptors, of which the type I receptor determines the specificity of biological responses by targeting different downstream signaling proteins and thus initiates distinct intracellular signaling cascades [2]. To determine which type I receptors are involved in the BMP2-induced upregulation of GREM2, we first used a pharmacological inhibition approach using different inhibitors of the type I receptor. We pretreated SVOG cells with DMSO (vehicle control), DM (a specific receptor kinase inhibitor of ALK2/ALK3/ALK6), DMH-1 (a selective inhibitor of ALK2/ALK3), or SB431542 (a selective inhibitor of ALK4/ALK5/ALK7) for 1 h prior to treatment with BMP2 for another 12 h. The results showed that DM or DMH-1 completely abolished the BMP2-induced upregulation of GREM2 (Fig. 2A and B), while pretreatment with SB431542 did not have such an effect (Fig. 2C). Thus, to further explore the specific type I receptor involved in this cellular activity, we used a siRNA-based depletion approach to knock down endogenous ALK2 and ALK3 in SVOG cells. The knockdown efficiency of specific ALKs was evaluated using RT-qPCR (Fig. 2D and E). Intriguingly, knockdown of ALK2 or ALK3 with siRNAs significantly attenuated the mRNA levels of GREM2 induced by BMP2 treatment (Fig. 2F and G). Consistent with the mRNA results, the BMP2-induced increase in the protein levels of GREM2 was significantly attenuated by the knockdown of ALK2 or ALK3 with siRNAs (Fig. 2H and I). These results indicate that both ALK2 and ALK3 are involved in the BMP2-induced upregulation of GREM2 in SVOG cells.

### SMAD1 and SMAD5 mediate the BMP2-induced upregulation of GREM2 in SVOG cells

**Fig. 3:**
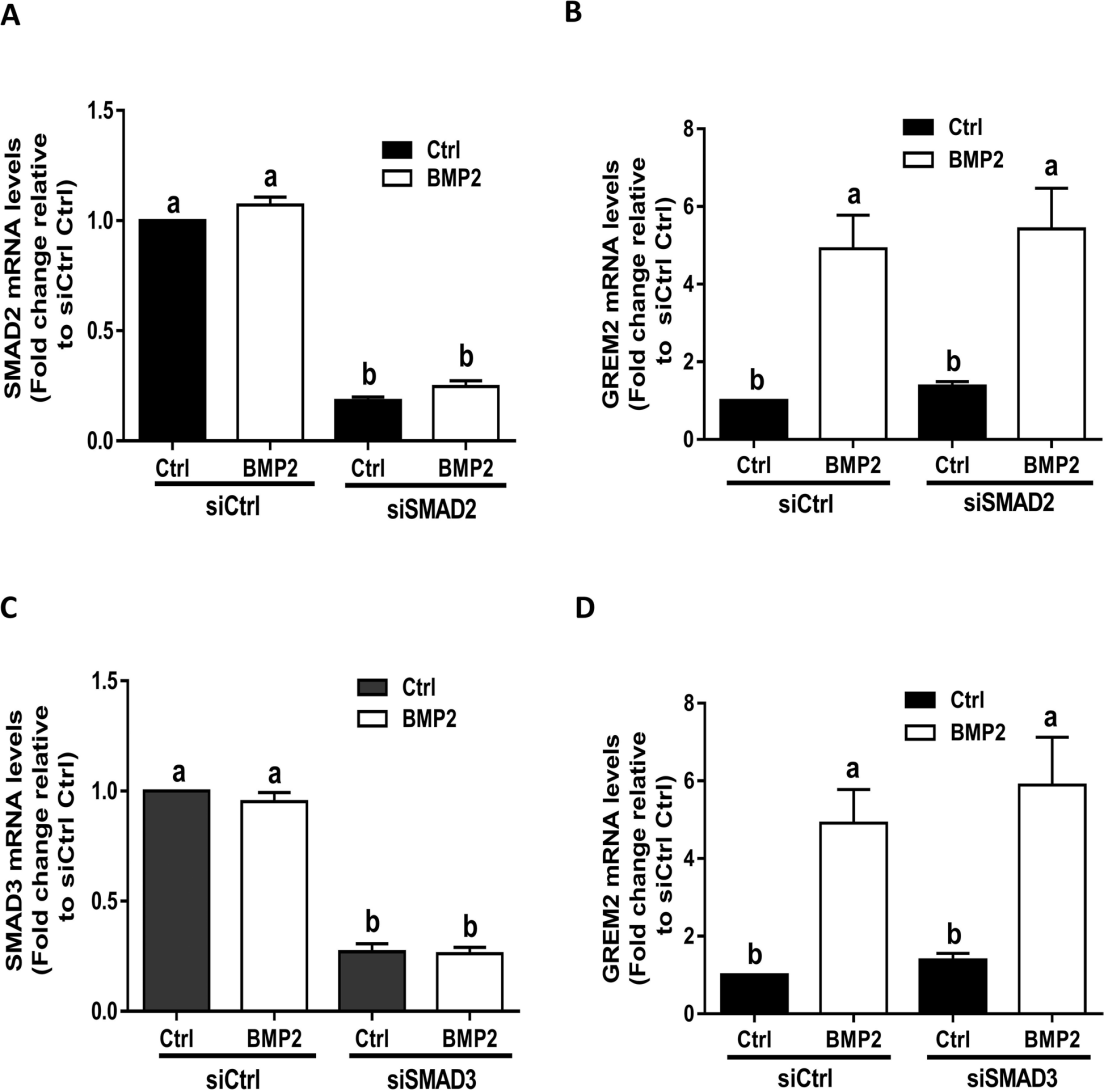
SMAD2 and SMAD3 do not mediate the BMP2-induced upregulation of GREM2 in SVOG cells. **A** and **B**, the effect of knockdown of SMAD2 on the BMP2-induced increase in the mRNA levels of SMAD2 and GREM2. **C** and **D**, the effect of knockdown of SMAD3 on the BMP2-induced increase in the mRNA levels of SMAD3 and GREM2. The results are expressed as the mean ± SEM of at least three independent experiments, and values without common letters are considered significantly different (*P* < 0.05)

**Fig. 4:**
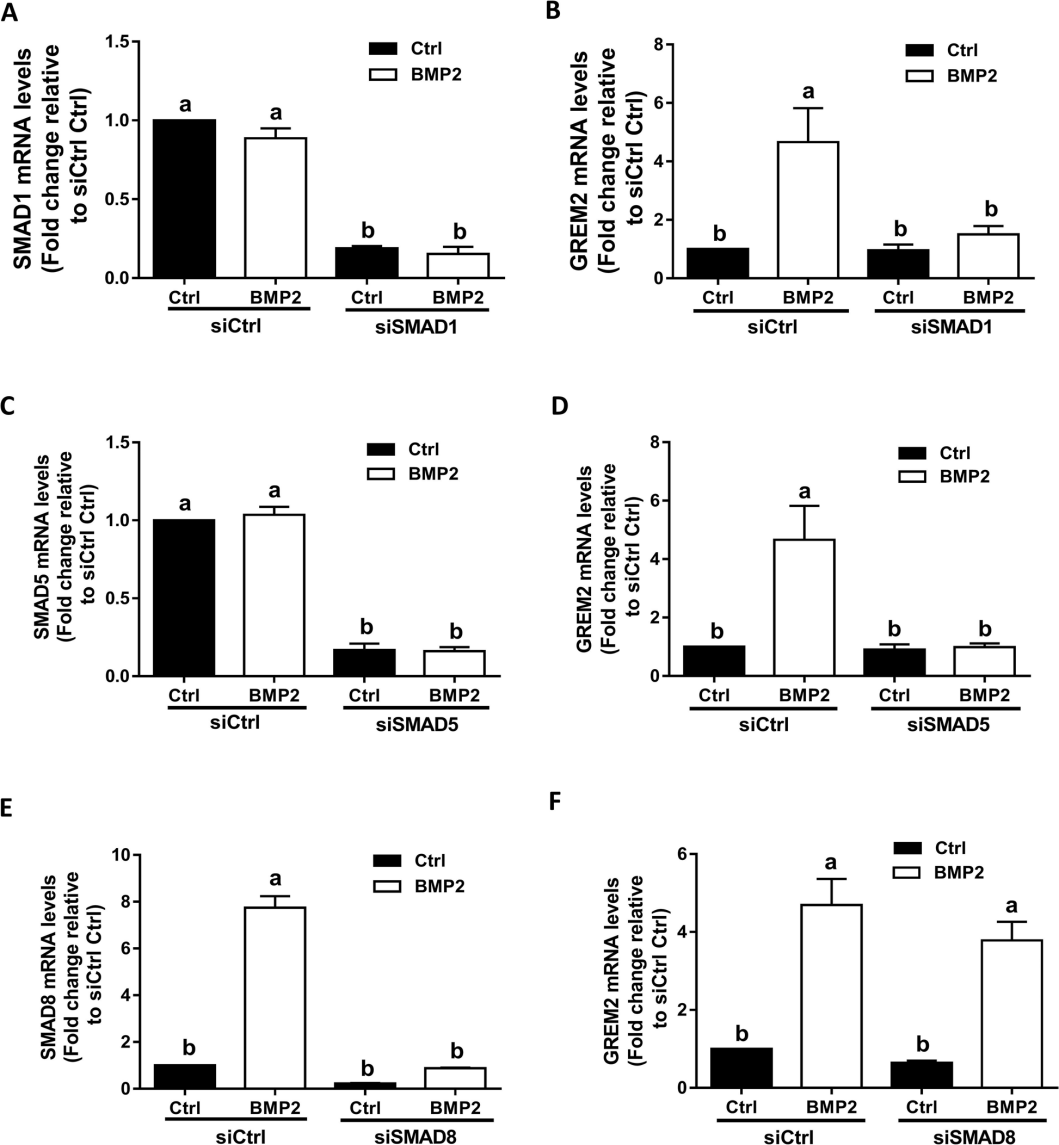
SMAD1 and SMAD5 mediate the BMP2-induced upregulation of GREM2 in SVOG cells. **A** and **B**, the effect of knockdown of SMAD1 on the BMP2-induced increase in the mRNA levels of SMAD1 and GREM2. **C** and **D**, the effect of knockdown of SMAD5 on the BMP2-induced increase in the mRNA levels of SMAD5 and GREM2. **E** and **F**, the effect of knockdown of SMAD8 on the BMP2-induced increase in the mRNA levels of SMAD8 and GREM2. The results are expressed as the mean ± SEM of at least three independent experiments, and values without common letters are considered significantly different (*P* < 0.05)

Upon binding to type I and type II receptors, BMPs regulate downstream target gene expression by phosphorylating receptor-regulatory SMAD (R-SMAD) transcription factors, including SMAD1/5/8 and SMAD2/3 [2]. To investigate which SMAD mediated the BMP2-induced upregulation of GREM2, we first used siRNA-mediated target depletion to knock down endogenous SMAD2 and SMAD3. RT-qPCR was used to evaluate the knockdown efficiency of each SMAD, and the results showed that the mRNA levels of each SMAD were significantly decreased by up to 80–90% 48 h after cell transfection (Fig. 3A and C). Notably, knockdown of either SMAD2 or SMAD3 did not alter the stimulatory effects of BMP2 on GREM2 expression (Fig. 3B and D). Similarly, we used the same approach to knock down endogenous SMAD1, SMAD5 and SMAD8. The knockdown efficiency of each targeted SMAD was confirmed using RT-qPCR (Fig. 4A, C and E). The results showed that knockdown of SMAD1 or SMAD5 (but not SMAD8) using siRNA completely abolished the mRNA levels of GREM2 in response to BMP2 (Fig. 4B, D, and F).

### SMAD4 mediates the BMP2-induced upregulation of GREM2 in SVOG cells

**Fig. 5:**
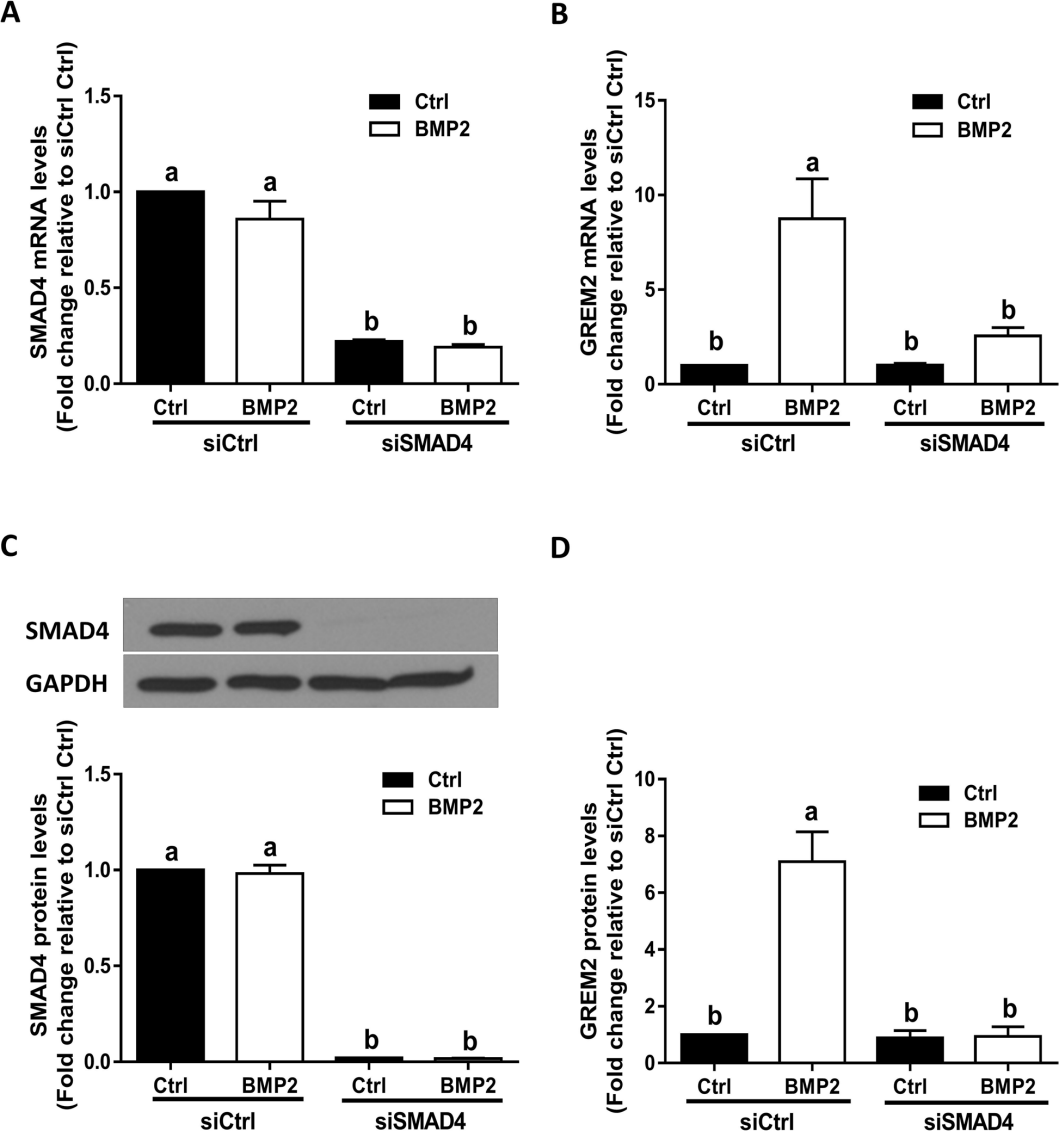
SMAD4 mediates the BMP2-induced upregulation of GREM2 in SVOG cells. **A** and **B**, the effect of knockdown of SMAD4 on the BMP2-induced increase in the mRNA levels of SMAD4 and GREM2. **C** and **D**, the effect of knockdown of SMAD4 on the BMP2-induced increase in the protein levels of SMAD4 and GREM2. The results are expressed as the mean ± SEM of at least three independent experiments, and values without common letters are considered significantly different (P < 0.05)

In the canonical SMAD-dependent pathway, phosphorylated R-SMADs form a heterotrimeric transcription factor complex with SMAD4 prior to translocating into the nucleus to regulate target genes. To further confirm the role of the SMAD signaling pathway in the BMP2-induced upregulation of GREM2, we transfected SVOG cells with siSMAD4. The knockdown efficiency of SMAD4 was verified using RT-qPCR and western blot analysis (Fig. 5A and C). Notably, the siRNA-mediated depletion of SMAD4 completely abolished the BMP2-induced increases in the mRNA (Fig. 5B) and protein (Fig. 5D) levels of GREM2.

### GREM2 suppresses the BMP2-induced phosphorylation of SMAD signaling and downregulation of StAR expression in primary and immortalized hGL cells

**Fig. 6:**
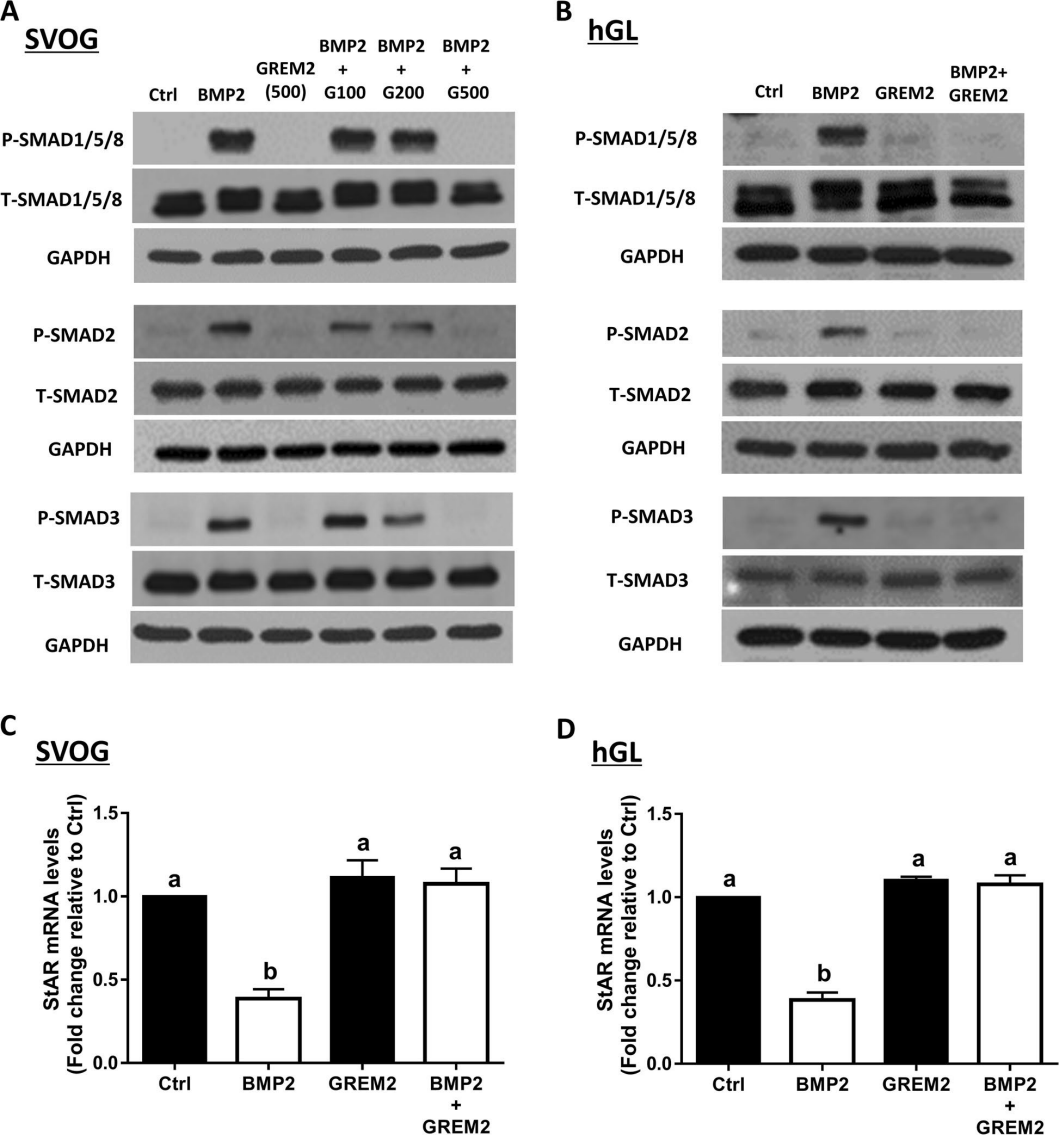
GREM2 suppresses the BMP2-induced increases in phosphorylated SMAD1/5/8, SMAD2 and SMAD3 and downregulation of StAR in primary and immortalized hGL cells. **A** and **B**, the effects of different concentrations of GREM2 on the BMP2-induced increases in the phosphorylated proteins of SMAD1/5/8, SMAD2 and SMAD3. **C** and **D**, the effects of GREM2 on the BMP2-induced decrease in the mRNA level of StAR in SVOG (**C**) and primary hGL (**D**) cells. The results are expressed as the mean ± SEM of at least three independent experiments, and values without common letters are considered significantly different (P < 0.05). StAR, steroidogenic acute regulatory protein

To investigate the suppressive effect of GREM2 on BMP2-induced cellular activity in hGL cells, we first treated SVOG cells with vehicle Ctrl, 50 ng/mL BMP2 or different concentrations (100, 200, or 500 ng/mL) of GREM2 for 1 h. The results showed that GREM2 decreased the phosphorylated protein levels of SMAD1/5/8, SMAD2, and SMAD3 in a dose-dependent manner in SVOG cells (Fig. 6A). To further confirm the biological effect of GREM2 in hGL cells, nonimmortalized primary hGL cells obtained from patients undergoing IVF treatment were used to perform the experiments. Similarly, the results showed that GREM2 at 500 ng/mL completely inhibited the protein levels of phosphorylated SMAD1/5/8, SMAD2, and SMAD3 in primary hGL cells (Fig. 6B). Steroidogenic acute regulatory protein (StAR) is the rate-limiting enzyme important for progesterone synthesis of granulosa cells that has been demonstrated to be downregulated by BMP2 in hGL cells [5]. We thus chose StAR as the target gene to investigate the effect of GREM2 on BMP2-induced cell function. The result showed that cotreatment with BMP2 and GREM2 completely reversed the BMP2-induced downregulation of StAR expression in SVOG cells (Fig. 6C). Similarly, cotreatment with BMP2 and GREM2 completely reversed the BMP2-induced downregulation of StAR expression in primary hGL cells (Fig. 6D).

### BMP2 suppresses GDF8-induced phosphorylation of SMAD2/3 in SVOG and primary hGL cells

**Fig. 7:**
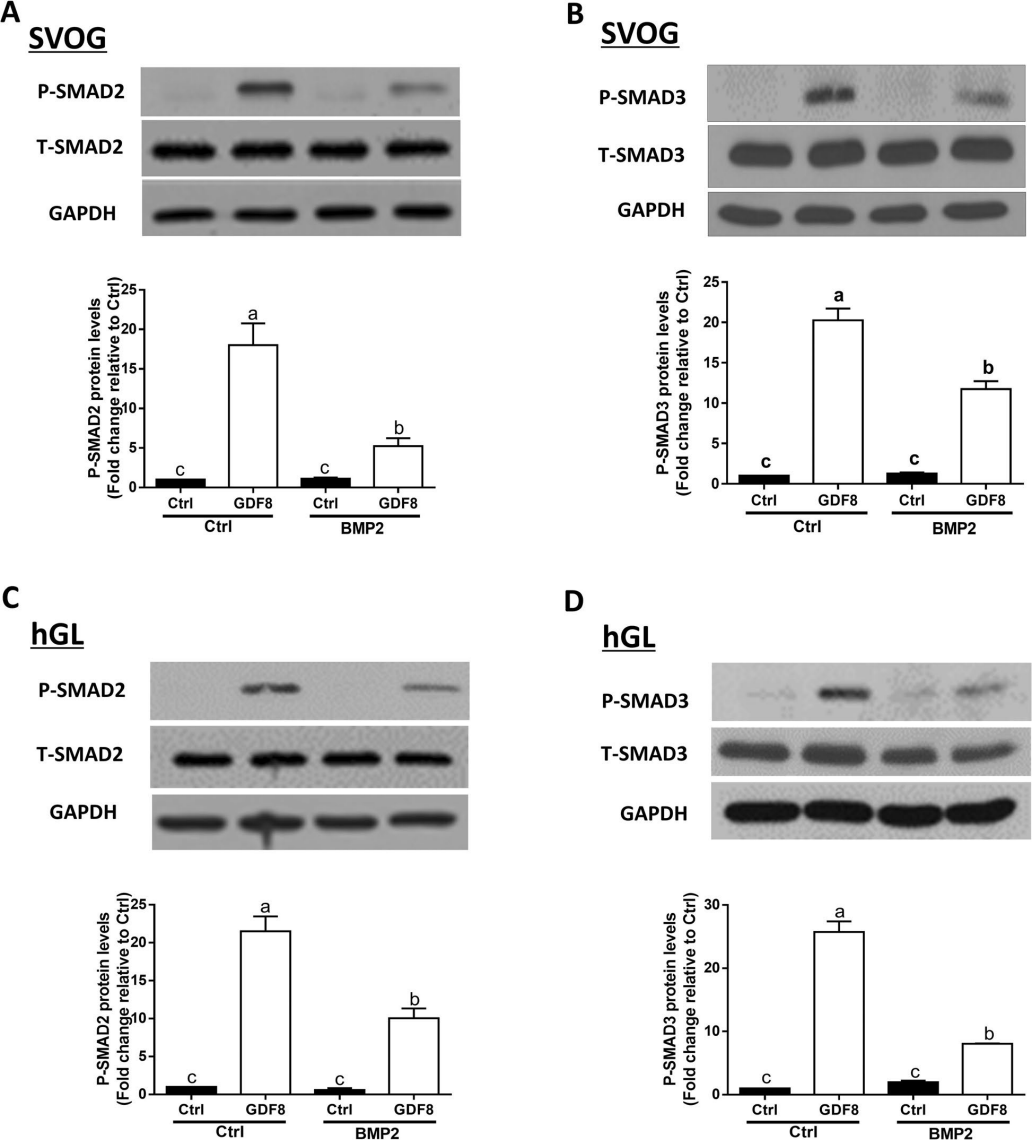
Pretreatment with BMP2 suppresses GDF8-induced phosphorylation of SMAD2/3 in SVOG and primary hGL cells. **A** and **B**, the effects of pretreatment of BMP2 on the GDF8-induced increases in the phosphorylated protein levels of SMAD2 (**A**) and SMAD3 (**B**) in SVOD cells. **C** and **D**, the effects of pretreatment of BMP2 on the GDF8-induced increases in the phosphorylated protein levels of SMAD2 (**A**) and SMAD3 (**B**) in primary hGL cells. The results are expressed as the mean ± SEM of at least three independent experiments, and values without common letters are considered significantly different (P < 0.05). GDF8, growth differentiation factor 8

To investigate whether BMP2 affected the GDF8-induced cellular activity in human granulosa cells, we pretreated SVOG cells with 50 ng/mL BMP2 for 24 h and then treated the cells with 30 ng/mL GDF8 for an additional 1 h. The results showed that pretreatment with BMP2 significantly attenuated the GDF8-induced increases in the protein levels of phosphorylated SMAD2 and SMAD3 in SVOG cells (Fig. 7A and B). Consistent with the results obtained from SVOG cells, pretreatment with BMP2 for 24 h significantly attenuated the GDF8-induced increase in the protein levels of phosphorylated SMAD2 and SMAD3 in primary hGL cells (Fig. 7C and D).

### GREM2 mediates the suppressive effects of BMP2 on the GDF8-induced increases in phosphorylated SMAD2 and SMAD3 in SVOG cells

**Fig. 8:**
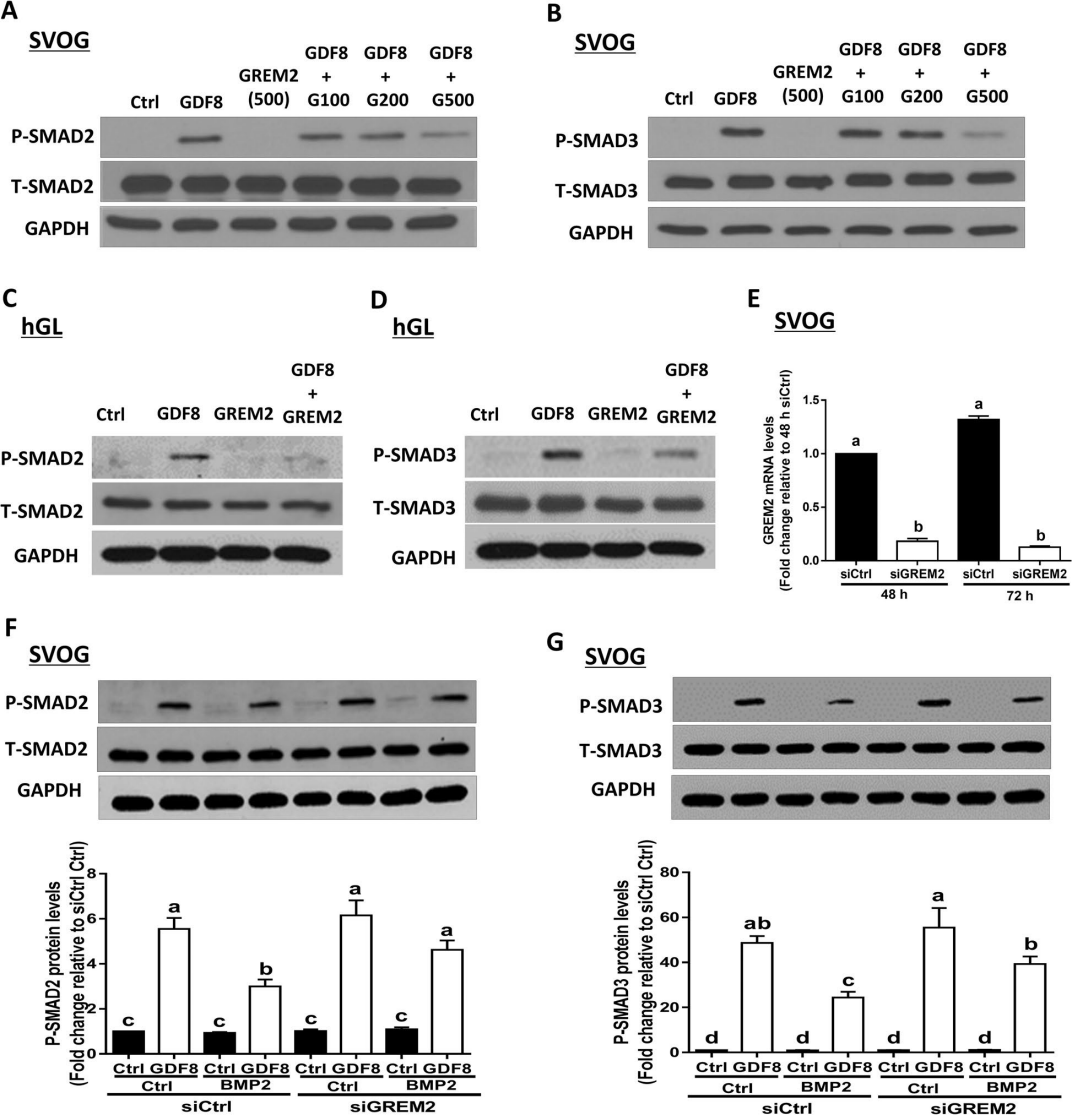
GREM2 mediates the suppressive effects of BMP2 on the GDF8-induced increases in phosphorylated SMAD2 and SMAD3 in SVOG cells. **A** and **B**, the effects of GREM2 on the GDF8-induced increases in the phosphorylated protein levels of SMAD2 (**A**) and SMAD3 (**B**) in SVOG cells. **C** and **D**, the effects of GREM2 on the GDF8-induced increases in the phosphorylated protein levels of SMAD2 (**C**) and SMAD3 (**D**) in primary hGL cells. **E**, the effects of knockdown of GREM2 on the mRNA levels of GREM2 in SVOG cells. **F** and **G**, GREM2 mediates the suppressive effects of BMP2 on the GDF8-induced increases in phosphorylated SMAD2 (**F**) and SMAD3 (**G**) in SVOG cells. The results are expressed as the mean ± SEM of at least three independent experiments, and values without common letters are considered significantly different (P < 0.05)

Given that BMP2 upregulates the expression of GREM2, we aimed to investigate the functional role of this effect in hGL cells. To investigate whether GREM2 antagonizes GDF8-induced cellular activity in hGL cells, we treated SVOG cells with vehicle Ctrl, 30 ng/mL GDF8, 500 ng/mL GREM2, or different concentrations (100, 200, or 500 ng/mL) of GREM2 for 1 h. The results obtained from western blot analysis showed that exogenous GREM2 decreased the GDF8-induced increases in the protein levels of phosphorylated SMAD2 and SMAD3 in a dose-dependent manner in SVOG cells (Fig. 8A and B). Similarly, the addition of GREM2 also significantly decreased the GDF8-induced increases in the protein levels of phosphorylated SMAD2 and SMAD3 in primary hGL cells (Fig. 8C and D). In this regard, we hypothesized that GREM2 is the intrafollicular factor that mediates the suppressive effects of BMP2 on GDF8-induced cellular activity in hGL cells. To test this hypothesis, we used a siRNA-mediated inhibition approach to knock down endogenous GREM2. Notably, transfection with 25 nM siGREM2 for 48 h and 72 h significantly decreased the mRNA levels of GREM2 in SVOG cells (Fig. 8E). Most importantly, the suppressive effects of BMP2 pretreatment on the GDF8-induced increases in SMAD2 and SMAD3 phosphorylation were significantly reversed by knocking down GERM2 expression in SVOG cells (Fig. 8F and G).

## Discussion

BMPs have been shown to alter the expression of various modulators involved in BMP signaling, including pseudoreceptors and antagonists, which provides negative feedback control in regulating BMP signaling [8, 43]. Our previous study showed that BMP2 upregulates the expression of BAMBI (a transmembrane pseudoreceptor antagonizing TGF-β/BMP signaling), which presents a negative feedback mechanism on BMP signaling in hGL cells [8]. Extracellular BMP antagonists are characterized by their direct protein-protein binding to BMP ligands through their cystine knots, thus subsequently preventing their interaction with receptors [44]. GREM1 and GREM2 are well-known extracellular BMP antagonists from the DAN family. In the present study, we demonstrated for the first time that BMP2 significantly increased the expression of GERM2 (but not GREM1) at both the transcriptional and translational levels in hGL cells. Previous studies have shown that treatment with BMP2 downregulates the expression of GREM1, while upregulates the expression of GREM2 in mouse myoblast (C2C12) cells [45]. In osteoblastic cells isolated from preosteoblasts of embryonic day 18.5 (E18.5) mouse calvariae, BMP2 was shown to increase the mRNA level of GREM2 [46]. In human fetal ovarian somatic cells, BMP2 promotes the upregulation of GREM1, whereas BMP2 does not alter the expression of GREM2 [43]. These discrepant results demonstrate that the regulation of the expression of GREM1 and GREM2 in response to BMP2 is species- and cell type-specific.

A comprehensive understanding of the molecular mechanisms involved in the BMP2-induced regulation of GREM2 expression will benefit us in developing new pharmacological strategies for clinical applications. BMPs initiate their cellular response by binding to TGF-β type I (also known as activin receptor-like kinase or ALK) and type II receptors, in which the type I receptor is the principal determinant of downstream signal specificity [47]. In general, BMPs, some GDFs, and AMH bind to ALK2/3/6, whereas TGF-βs, other GDFs, activins, and nodal bind to ALK4/5/7 [2]. However, this notion has been challenged by recent studies showing that BMP2 can also bind to ALK4/5/7 and activate downstream signaling [48, 49]. To investigate which TGF-β type I receptor mediated the BMP2-induced upregulation of GREM2, we used a dual inhibition approach. We first pretreated SVOG cells with different TGF-β type I receptor inhibitors, including DM (inhibitor of ALK2/3/6), DMH-1 (inhibitor of ALK2/3), and SB431542 (inhibitor of ALK4/5/7) for 1 h prior to BMP2 administration. The results showed that DM or DMH-1 (but not SB431542) completely abolished BMP2 induced upregulation of GREM2, indicating that either ALK 2 and/or ALK3 type I receptors were involved in this cellular activity in hGL cells. To further determine which specific ALK (ALK2 or ALK3) mediated the BMP2-induced upregulation of GREM2, we knocked down endogenous ALK2 or ALK3 using a siRNA-mediated target depletion approach. The results showed that the knockdown of either ALK2 or ALK3 completely abolished the BMP2-induced increases in the mRNA and protein levels of GREM2. Taken together, these findings indicate that the upregulation of GREM2 induced by BMP2 is mediated by ALK2 and ALK3 type I receptors.

In canonical SMAD-dependent signaling, phosphorylated ALK2/3/6 activate BMP-induced SMAD1/5/8 signaling, while phosphorylated ALK4/5/7 activate TGF-β/activin-induced SMAD2/3 signaling [2]. However, in addition to SMAD1/5/8, our recent studies have shown that BMP2 also activates SMAD2/3 signaling via ALK3. In this study, we showed that although ALK3 was involved in the BMP2-induced upregulation of GREM2 expression, the targeted depletion of SMAD1, SMAD5, or SMAD4 completely abolished the increase in GREM2 mRNA levels. However, the targeted depletion of SMAD2, SMAD3, or SMAD8 did not have such an effect. These findings indicate that ALK2/3-SMAD1/5-SMAD4 is the principal downstream signaling pathway that mediates the BMP2-induced upregulation of GREM2 in hGL cells.

Given that GREM2 is a potent antagonist of BMP2, and the BMP-binding epitope on GREM2 is located at the hydrophobic interface on its convex surface (beneath the α1 helix in the DAN domain), through which GREM2 directly binds to the type I and type II receptor-binding epitopes for BMPs and inhibits the BMP signaling [44], we first investigated the effect of GREM2 on BMP2-induced cellular signaling in hGL cells. Our results showed that the addition of GREM2 suppresses BMP2-induced increases in the protein levels of phosphorylated SMAD2, SMAD3, and SMAD1/5/8 in a dose-dependent manner. Moreover, addition of GREM2 completely reversed BMP2-induced downregulation of StAR expression, further demonstrated the antagonistic effect of GREM2 on BMP2-induced cell function. To date, most GREM2-related studies have focused on its antagonistic effects on BMP-induced cellular activity, especially BMP2 and BMP4. Only a handful of studies have investigated the effect of GREM2 on other TGF-β superfamily members, and the results are not consistent. One study carried out in 293 T cells showed that GREM2 significantly antagonizes the stimulation of promoter-luciferase activities induced by BMP2, BMP4, BMP6, and BMP7, while GREM2 does not affect the reporter activity induced by activin, TGF-β, GDF9 and GDF5 [23]. However, another study demonstrated that the GREM2 dimer can bind to each GDF5 monomer in a perpendicular manner and form a stable aggregate-like structure with appreciable affinity [50]. GDFs and BMPs share many overlaps in their structures, and some antagonists (such as follistatin) exert suppressive effects on both factors [51]. However, to the best of our knowledge, no reports have investigated the antagonistic effect of GREM2 on GDF8-stimulated cellular activity in any animal model. In this study, we examined the effects of GREM2 on GDF8 induced cell signaling in hGL cells. The results showed that GREM2 significantly attenuated the GDF8-induced increases in SMAD2 and SMAD3 phosphorylation in a dose-dependent manner. Additionally, pretreatment with BMP2 attenuated the GDF8-induced phosphorylation of SMAD2 and SMAD3 in hGL cells. Moreover, knockdown of GREM2 reversed the BMP2-induced suppressive effect on GDF8-induced increases in SMAD2/3 phosphorylation. These results indicate that BMP2 upregulates the expression of GREM2 and that the increased GREM2 in turn antagonizes GDF8-induced cellular signaling in hGL cells. Our novel findings suggest that in addition to BMPs, GREM2 may also antagonize another critical intraovarian factor (GDF8) in hGL cells. However, future structural analysis studies regarding the molecular interaction between GREM2 and GDF8 will be of great interest.

## Conclusions

In conclusion, our study demonstrated that BMP2 upregulates the expression of GREM2 via the ALK2/3-SMAD1/5-SMAD4-dependent signaling pathway. Notably, we provided the first data showing that GREM2 directly antagonizes the cellular activity induced by GDF8 and that GREM2 mediates the BMP2-induced suppressive effects on GDF8-induced cellular response in hGL cells. These findings deepen our understanding of the interactions among these growth factors in the human ovary, which may potentially help develop targeted therapeutic approaches for women with reproductive disorders in the future.

## Data Availability

All data generated or analyzed during this study are included in this published article.

## Abbreviations

ALK: Activin receptor-like kinase
BAMBI: Bone morphogenetic protein and activin membrane-bound inhibitor
BMP: Bone morphogenetic protein
BSA: Bovine serum albumin
DM: Dorsomorphin dihydrochloride
DAN: Differential screening-selected gene in neuroblastoma
DMH-1: 4-[6-[4-(1-methylethoxy) phenyl] pyrazolo [1, 5-a] pyrimidin-3-yl]-quinoline
ELISA: Enzyme-linked immunosorbent assay
FSH: Follicle stimulating hormone
GAPDH: Glyceraldehyde-3-phosphate dehydrogenase
GDF: Growth differentiation factor
GREM1: Gremlin1
GREM2: Gremlin2
HCG: Human chorionic gonadotropin
hGL: Human granulosa-lutein
IVF: in vitro fertilization
LH: Luteinizing hormone
MMLV: Moloney murine leukemia virus
PBS: Phosphate-buffered saline
PVDF: Polyvinylidene difluoride
RT-qPCR: Reverse transcription quantitative real-time PCR
siRNA: small interfering RNA
SMAD: Sma- and Mad-related protein
StAR: Steroidogenic acute regulatory protein
TBS: Tris-buffered saline
TBST: Tris-buffered saline containing 0.1% Tween 20
TGF-β: Transforming growth factor-β
